# Cyberterrorism as a global threat: a review on repercussions and countermeasures

**DOI:** 10.7717/peerj-cs.1772

**Published:** 2024-01-15

**Authors:** Saman Iftikhar

**Affiliations:** Faculty of Computer Studies, Arab Open University, Riyadh, Saudi Arabia

**Keywords:** Cyberterrorism, Cyberattacks, Networking, Security, Political instability, Nation’s economy

## Abstract

An act of cyberterrorism involves using the internet and other forms of information and communication technology to threaten or cause bodily harm to gain political or ideological power through threat or intimidation. Data theft, data manipulation, and disruption of essential services are all forms of cyberattacks. As digital infrastructure becomes more critical and entry barriers for malicious actors decrease, cyberterrorism has become a growing concern. Detecting, responding, and preventing this crime presents unique challenges for law enforcement and governments, which require a multifaceted approach. Cyberterrorism can have devastating effects on a wide range of people and organizations. A country’s reputation and stability can be damaged, financial losses can occur, and in some cases, even lives can be lost. As a result of cyberattacks, critical infrastructure, such as power grids, hospitals, and transportation systems, can also be disrupted, leading to widespread disruptions and distress. The past ten years have seen several cyber-attacks around the globe including WannaCry attack (2017), Yahoo data breaches (2013–2014), OPM data breach (2015), SolarWinds supply chain attack (2020) *etc*. This study covers some of the cyberterrorism events that have happened in the past ten years, their target countries, their devastating effects, their impacts on nation’s economy, political instability, and measures adopted to counter them over the passage of time. Our survey-based research on cyberterrorism will complement existing literature by providing valuable empirical data, understanding of perceptions and awareness, and insights into targeted populations. It can contribute to the development of better measurement tools, strategies, and policies for countering cyberterrorism.

## Introduction

Cyberterrorism refers to the utilization of internet, information mediums and communication platforms to conduct terrorist attacks or to promote terrorist causes. These attacks can take many forms, such as disseminating propaganda, stealing or manipulation of data, or disrupting critical infrastructure. It is also possible to refer to it as an act of unauthorized attacks and threat-making against computers, networks, and the data they house and disseminate (Theohary & Rollins, 2015). To achieve a political or social goal, this is done through intimidating or threatening a government or its citizens. High intensity cyberattacks can also inflict violence against persons or property, or at the very least enough damage to inspire fear. Most of the time, cyberterrorism can result in death or physical harm, an explosion, a plane accident, water pollution, or a major economic or political loss. If it has a large effect, cyberterrorism may be conducted against essential infrastructure. Attacks that interfere with unnecessary services or are merely a nuisance do not need to be reported. Cyberterrorism is the deliberate use of cyber capabilities, often by non-state actors, with the primary intention of causing widespread fear, panic, or disruption in a population, government, or organization. Acts of cyberterrorism typically involve politically, ideologically, or socially motivated attacks that target critical infrastructure, result in significant harm, or pose a grave threat to national security. What distinguishes cyberterrorism from other cyberattacks, such as cybercrime or hacktivism, is the explicit intent to incite terror or destabilize societies, often in pursuit of political or ideological goals, rather than purely financial gain or the pursuit of social or ethical objectives. Cyberterrorism seeks to create fear, chaos, and mistrust on a larger scale, often with the potential for real-world harm or destruction.

This idea is not sufficiently understood. Recent attempts to broaden the definition of cyberterrorism to encompass hacktivism and the use of the Internet by terrorists to further conventional terrorism is primarily to blame for the uncertainty around the term. The biggest online threat posed by a non-state terrorist group comes from their capacity to use the Internet for purposes other than cyber-terrorism, such as fund-raising, target research, and supporter recruitment. Although cyber-terrorism may arise in the future, online crime, hacktivism, and cyber-warfare pose more immediate threats (Kenney, 2015). A striking feature of our understanding of cybercrime is the variety of terms used to describe it. Despite the wide range of terminology used, there is one common thread that stands out. In earlier ways of thinking about misuse of information technology, this was called ‘crime by computer.’ Pre-Internet, computers were the primary target of crime, so this seemed like an appropriate name. Even though networked computing became widespread in the 1990s, this term has continued to be used. Until 2000, it was the most used term for crimes related to information technology. Other words, such as e-crime, online crime, digital crime, net crime, techno-crime, Internet crime, or even hi-tech crime, have all been used at various points in time. In the past, the phrase used to characterize the crime was computer crime. Academic literature on cybercrime contains two times as many references to this term. However, by 2018, the situation had significantly changed. In scholarly sources between 2001 and 2018, there were twice as many references to cybercrime as to ‘computer crime’, making cybercrime the preferred term of choice (Kapto, 2013). There has been a cybercrime problem for more than three decades in various forms. As technology has become more widely used and its criminal potential has become more widely recognized, some forms of cyber-attack reported by industry seem to have been increasing in scale and breadth. Public awareness has also increased, as has recognition by governments, businesses, and legal systems. It has been difficult to accurately measure cyber-crime scale and trends (not just attacks), or assess the harms and impacts caused by successful attacks (Furnell & Dowling, 2019). Cyberwarfare is the umbrella word for attacks on and defenses against computer networks, as well as unique technological activities. Cyberwarfare is the term used to describe when a country utilizes digital attacks, such as computer viruses and hacking, to damage, kill, and destroy another country’s critical computer systems. In the future, hackers will fight alongside traditional weaponry like guns and missiles, attacking an adversary’s infrastructure using computer code. Cyberwarfare has emerged as a regular and deadly facet of international conflict in a world still filled with spies, hackers, and top-secret digital weapons programs. However, now there is a genuine risk of situations quickly spiraling out of control due to the continuous weapons competition in cyberwarfare and the absence of defined guidelines governing online combat (Ranger, 2018).

Cyberterrorism is a growing concern because of the increasing reliance on information technology in many aspects of society and the potential for significant disruption or harm caused by cyber-attacks. It had a significant impact globally and continues to threaten the various aspects of a nation’s stability. One of the biggest effects of cyberattacks is the interruption of vital infrastructure, such as transportation networks, power grids, and banking networks. Directed attacks on these systems can have widespread consequences including blackouts, transportation delays, and financial losses (Caplan, 2013). In addition, cyberterrorism has been used to spread propaganda and manipulate public opinion, as well as to steal sensitive information, such as intellectual property or personal data. The fear of cyberterrorism has also led to increased spending on cybersecurity measures by governments and private companies. The world has been affected in several ways by the act of cyber-attacks including infrastructural damage, monetary crisis, economic crisis, the spread of propaganda or misinformation, loss of sensitive information, and privacy invasion etc. Cyberterrorism can lead to significant financial losses for businesses and governments, as well as damage to a country’s reputation and economic stability. It can be used to spread propaganda and manipulate public opinion, which can lead to social and political instability. Cyber-attacks can result in the theft of sensitive information, such as intellectual property or personal data, which can have profound consequences for individuals and organizations. The fear of getting engaged in the vicious act of cyber-invasion has led to increased spending on cybersecurity measures by governments and private companies. Cyberattacks can result in the loss of privacy, as personal data is stolen or made public (Foltz, 2004). Overall, cyberterrorism can cause significant disruption and harm, both to individuals and society. This survey paper is intended for a wide range of audiences, including businesses, companies, government bodies and individuals to get a varied awareness about all these threats to them.

Our survey-based research on cyberterrorism will make unique contributions to the existing literature in several ways. It will provide a structured means of collecting empirical data on various aspects of cyberterrorism. This data can offer insights into the prevalence, patterns, and motivations of cyberterrorist activities, which may not be as readily available through other research methods. Moreover, public or expert perceptions and awareness of cyberterrorism can help assess how different groups perceive the threat, its severity, and the measures they believe are necessary to counter it. It will develop and refine measurement tools and metrics specific to cyberterrorism. This can lead to more accurate assessments of its impact and effectiveness in different contexts. Researchers can target specific populations or groups, such as cybersecurity professionals, government officials, or the public. This targeted approach can provide valuable insights into the views and experiences of these groups regarding cyberterrorism. They can also conduct comparative analyses by collecting data from multiple sources or over time. This can help track changes in attitudes, awareness, and perceptions related to cyberterrorism. This research can reveal vulnerabilities and preparedness levels among organizations, governments, or individuals. This information can be crucial for developing effective strategies to counter cyberterrorism. In addition to quantitative data, surveys can include open-ended questions, which provide qualitative insights into the nuanced perspectives and experiences related to cyberterrorism. These results can inform the development of policy recommendations and strategies for governments, organizations, and other stakeholders in their efforts to prevent and respond to cyberterrorism. In Addition, risk assessment can be done by examining the perceived and actual risks associated with cyberterrorism. This can help prioritize resources and responses. Finally, public opinion can be revealed on issues related to cyberterrorism, which can influence political will and shape government policies and actions in response to the threat.

### Motivation behind survey

Lack of comprehensive, actual-world case studies is one gap in the cyberterrorism literature and study, currently in existence. While several studies have examined the general trends and patterns of cyberattacks, in-depth examination of occurrences is lacking. This makes it challenging to completely comprehend the complexity of various cyberattacks and the unique difficulties that governments and businesses have in reacting to them. The inadequate attention paid to the effects of cyberterrorism is another flaw in the literature and study that has already been done. Less study has been done on the long-term impacts of cyberattacks on people, organizations, and society, even though there is a lot of literature on many types of cyberattacks that have been conducted and the countermeasures that have been used. This encompasses the effects of cyberterrorism on the economy, the mind, and society. It is critical to comprehend these effects to create effective defenses and evaluate the overall effect of cyberattacks. The amount of research on the countermeasures used by nations is equally limited, necessitating more thorough analyses of their efficacy.

To ensure a comprehensive and unbiased analysis of the topic of cyberterrorism, the literature and studies used in this paper were selected from a variety of reputable sources, including academic journals, government reports, and news articles. The sources were carefully chosen to ensure that they covered a wide range of perspectives and viewpoints on the topic. Additionally, the literature review and case studies were selected based on their relevance to the research question and their availability of detailed information. The literature review was performed by considering studies from different regions (Middle East, East Asia, USA, Russia, France) and covering different periods (last 10 to 15 years). Furthermore, an effort was made to ensure that the countermeasures adopted by various countries were evaluated in a neutral manner, considering the effectiveness of the measures and not the political background of the countries. All sources were critically evaluated to ensure that they were credible and unbiased.

### Survey methodology

**Figure 1:**
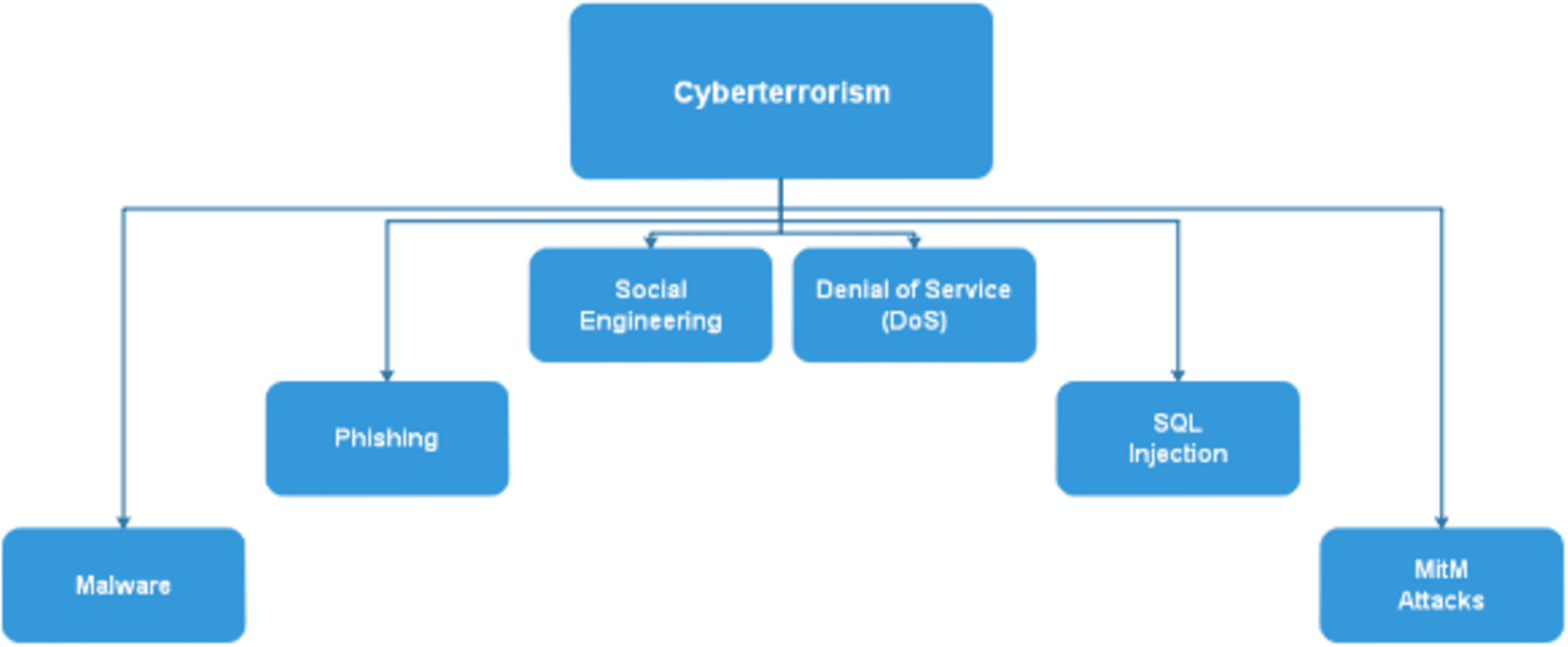
Various methods through which cyberterrorism is carried out.

The survey methodology used in this paper consisted of a thorough review of literature on the topic of cyberterrorism and its repercussions, as well as countermeasures adopted by various nations, regions, states, and countries. The literature review covered a range of sources, including academic journals, latest survey papers, government reports, and news articles. The focus of the literature review was on instances of cyberterrorism that have occurred in the Middle East, East Asia, USA, Russia, and France over the past 10-15 years. The literature review aimed to identify the key trends and patterns in terms of the types of cyberattacks that have been carried out, the sectors that have been targeted, and the repercussions of these attacks. In addition to the literature review, the study also included a qualitative analysis of case studies of specific cyberterrorism incidents that have occurred in the mentioned regions and around the globe. The studies, papers, articles, and other sources considered for this article were selected based on their relevance to the research question and their availability of detailed information and were analyzed in terms of the types of attacks, the sectors targeted, the repercussions, and the countermeasures that were adopted. Finally, the study also included a review of the countermeasures adopted by various countries against future cyber-attacks. The review of countermeasures looked at the various organizations, sectors, agencies, strategies, and technologies that have been created, developed, and implemented by countries in the mentioned regions and around the world to protect against cyberattacks. The review aimed to identify the most effective countermeasures and to identify any gaps in the existing strategies. Overall, the survey methodology used in this paper aimed to provide a comprehensive and in-depth understanding of the topic of cyberterrorism, its repercussions, and the countermeasures adopted by various countries. The combination of literature review, case studies, and review of countermeasures provided a holistic view of the topic, highlighting the key trends, patterns, and gaps in the existing research. Figure 1. shows some of the methods through which cyberterrorists carry out the vicious act of cyberterrorism. Some of the common methods through which cyberterrorism is carried out include:

**Malware:** Malicious software, such as viruses, worms, Trojans, and ransomware, can be used to compromise computer systems and steal sensitive information, disrupt critical infrastructure, or create chaos. Cyberterrorists may develop or deploy malware to achieve their objectives.

**Phishing:** Phishing attacks involve the use of deceptive emails, websites, or messages to trick individuals into revealing sensitive information like login credentials, financial details, or personal data. These tactics can be used to gather intelligence or access critical systems.

**Denial of Service (DoS) and Distributed Denial of Service (DDoS) attacks:** DoS and DDoS attacks involve overwhelming a target’s computer systems or network with excessive traffic, causing them to become unavailable. Cyberterrorists may use these attacks to disrupt the operation of critical infrastructure or services.

**Social engineering:** Social engineering techniques involve manipulating individuals into revealing confidential information or performing actions that may compromise security. Cyberterrorists may impersonate trusted individuals or entities to gain access to sensitive data or systems.

**SQL injection:** SQL injection attacks target vulnerabilities in web applications that use SQL databases. Cyberterrorists can exploit these vulnerabilities to access, manipulate, or exfiltrate data from databases, potentially causing significant damage.

**Man-in-the-Middle (MITM) attacks:** MITM attacks intercept and alter communications between two parties, often without their knowledge. Cyberterrorists can use MITM attacks to eavesdrop on sensitive information, manipulate messages, or compromise the security of communication channels.

**Ransomware:** Ransomware is a type of malware that encrypts a victim’s data, making it inaccessible until a ransom is paid. Cyberterrorists may deploy ransomware to disrupt critical systems or extort money from targeted organizations.

**Insider threats:** Insider threats involve individuals within an organization who intentionally or unintentionally aid cyberterrorists in their activities. These individuals may have access to critical information or systems.

**Stuxnet-Like attacks:** Stuxnet is a famous example of a targeted cyberattack that specifically aimed at disrupting industrial control systems, such as those used in nuclear facilities. Cyberterrorists might target critical infrastructure systems to cause physical harm or destruction.

**Zero-Day exploits:** Cyberterrorists may employ unknown vulnerabilities in software or hardware systems known as zero-day exploits to gain unauthorized access or control over systems. These vulnerabilities are typically undisclosed to the software vendor or the public.

Cyberterrorists often use a combination of these methods to achieve their goals, and their motivations can vary widely, including political, ideological, financial, or simply causing chaos and disruption. It is crucial for individuals, organizations, and governments to implement strong cybersecurity measures to defend against cyberterrorism and its various tactics.

The selection of cyberterrorism events involves a multifaceted approach that combines methods such as incident reporting, threat intelligence, attribution analysis, open-source information, government reports, academic studies, international collaboration, legal frameworks, classification tools, expert consultations, historical analysis, and government threat assessments. These methods collectively aid in identifying and categorizing events that align with specific criteria, including significance, intent, targeting, and impact. However, event selection may entail a degree of subjectivity and interpretation, depending on the research objectives and the availability of data and resources. The selection criteria for cyberterrorism events encompass attributes such as the event’s significance in terms of damage or threat, clear attribution to a cyberterrorist entity, a political or ideological motive, targeted critical infrastructure or national security interests, specific methodologies like malware or DDoS attacks, the intent to cause fear or panic, coordinated efforts by a group, the scope and scale of impact, geopolitical context, adherence to legal definitions, and the potential for interpreting the actors’ intent based on available evidence. However, the selection process is hindered by challenges including attribution difficulties, subjective motive interpretation, underreporting, discrepancies in event classification, the evolving nature of cyberterrorism tactics, and potential bias or political influence.

### Impact of cyberterrorism on nation’s economy

Cyber-attacks can have a significant impact on a country’s economy. Some of the ways in which cyber-attacks can impact a country’s economy include direct financial losses, decreased productivity, increased budget for installing security measures against invasion, financial theft, and damaged reputation in front of the entire world. Cyber-attacks can result in direct financial losses for businesses and governments, as well as damage to a country’s reputation and economic stability. These invasions can disrupt the operation of businesses and organizations, leading to decreased productivity and lost revenue. Governments and businesses may need to spend more on cybersecurity measures to protect themselves from future attacks. The theft of sensitive information, such as intellectual property or trade secrets, can harm a country’s economy by giving competitors an advantage. can damage a country’s reputation, leading to a loss of trust and confidence in its businesses and institutions (Hua & Bapna, 2013). This may affect the nation’s capacity to draw foreign investment and travelers. In general, cyber-attacks can have major effects on a nation’s economy, hence it is crucial for governments and corporations to take precautions against these risks. Most significantly, when a nation is a victim of a significant cyberattack, its reputation suffers, which causes people to lose faith in its institutions and companies. This can impact the country’s ability to attract investment, which is an important source of revenue for many countries. There have been several instances in which cyber-attacks have contributed to economic crises. In 2017, the NotPetya ransomware attack (Fayi, 2018) affected businesses and government agencies in Ukraine and other countries, leading to direct financial losses and decreased productivity. The attack was estimated to have cost businesses billions of dollars. In 2017, the WannaCry ransomware attack (Mohurle & Patil, 2017) affected businesses and government agencies in more than 150 countries, leading to direct financial losses and decreased productivity. The attack was estimated to have cost businesses billions of dollars. SolarWinds, a significant supplier of IT monitoring software, was found to have been infiltrated (Wolff, Growley & Gruden, 2021) in 2020, allowing a group of hacker’s access to the networks of SolarWinds’ clients. Numerous companies and government entities in the US and worldwide were impacted by the attack, which resulted in immediate financial losses and lost productivity. The historical nature of cybercrime in Pakistan (Akram, Mir & Rehman, 2023) reveals two main types of cyberattacks: low-level attacks from individual Indian hackers and attacks from both local and international hackers seeking financial gains. One significant incident involved the hacking of Meezan Bank, resulting in the exposure of 69,189 card details for sale and causing approximately $3.5 million in data loss for the bank. Additionally, K-electric experienced a security breach, with hackers demanding a $3.5 million ransom. However, the ransom doubled to $7 million after a week, but K-electric did not comply, leading to the leaked online sale of stolen information, including sensitive customer data such as names, addresses, CNIC, and bank account details. Despite the severity of the situation, K-electric did not pay the ransom nor take sufficient measures to improve their cybersecurity, ultimately resulting in the hacker leaking 8.5 GB of data. These incidents highlight the urgent need for better cybersecurity measures in Pakistan to protect against such threats and safeguard sensitive information.

### Role of cyberterrorism in political instability

Cyberterrorism can also have a significant impact on politics in several ways including disruption of elections, spread of misinformation, propaganda, invasion of state secrets and privacy eruption. Cyberattacks on election systems or campaigns can sabotage the electoral process and erode public trust in the fairness of elections. Cyber terrorists may use the internet and social media to spread propaganda and manipulate public opinion, which can influence political events and shape public policy. Cyber-attacks on communication systems, such as email or phone networks, can hinder the ability of political leaders and organizations to communicate with each other and with the public. Cyber-attacks that result in the loss of privacy can undermine trust in political leaders and institutions (Weimann, 2005). Overall, cyberterrorism can have profound consequences for the political landscape of a country and can contribute to social and political instability. If the purpose of attackers is to disrupt elections, attacks on voting systems or infrastructure can disrupt the voting process and prevent people from casting their ballots. The culprits may spread misinformation or propaganda through social media and other online platforms to influence the outcome of elections. Attacks on political campaigns can disrupt campaign operations and compromise sensitive campaign data. Invasion of election systems or campaigns can undermine trust in the electoral process and lead to a loss of confidence in the integrity of elections. Cyber terrorists may use the internet and social media to spread propaganda and manipulate public opinion, which can lead to social and political instability and potentially the collapse of a government (Golase, 2022). They can result in the loss of privacy or sensitive information thus undermining trust in a government and can potentially lead to its collapse. It is difficult to say with certainty whether any government has fallen solely because of cyberterrorism, as most major events have multiple causes. However, there have been several instances in which cyber-attacks have played a role in social and political instability and the downfall of governments. In 2011 (Rahimi, 2011), a series of protests and uprisings swept across the Middle East and North Africa, leading to the overthrow of several governments. The use of social media to organize and disseminate information played a significant role in the Arab Spring, and some experts believe that cyber-attacks on government communication systems may have contributed to the instability. During the 2016 US presidential election (Berghel, 2017), Russian hackers were found to have targeted the campaign of Hillary Clinton and hacked the email accounts of Democratic Party officials, leading to the release of sensitive information through WikiLeaks. Some experts believe that these attacks may have influenced the outcome of the election. In 2020 (Voltz, 2021), it was discovered that a group of hackers had compromised the software of SolarWinds, a major provider of IT management software, to gain access to the networks of SolarWinds’ customers. The attack affected several government agencies in the United States, leading to concerns about the integrity of the US government’s systems and the potential for further damage. The concept of cyberterrorism can be traced back to the 1990s (Baldassarre, 2023a), when the rise of the Internet and discussions on the “information society” raised concerns about potential risks for the highly networked US. This notion of cyberterrorism evoked psychological fear, combining apprehension of random violence with distrust of computer technology. After the 9/11 attacks, cyberterrorism gained prominence in security and terrorism discourse, and debates over national security attracted political actors with broader agendas. The media played a role in sensationalizing cyberterrorism, leading to misuses of the term and overblown reactions to incidents, which muddled the understanding of the actual threat posed by cyberterrorism.

The post-9/11 era (Bastug, Onat & Guler, 2023) saw an emergence of a lucrative industry dedicated to countering cyberterrorism, with think tanks, experts, and private companies actively addressing the issue. Government investment and public concern heightened, with warnings from high-level officials and media coverage further fueling anxiety. However, this climate of heightened attention has led to instances of labeling hacking and cybercrimes as “cyberterrorism” without a precise definition. To better grasp the true danger of cyberterrorism, it is essential to define the term accurately and distinguish between actual threats and broader concerns about cybersecurity. In summary, the concept of cyberterrorism emerged in the 1990s, evoking psychological fear and becoming a focal point after the 9/11 attacks. The subsequent years witnessed both political and economic investments in countering cyberterrorism, along with media sensationalism that muddled the understanding of the actual threat. A clear and precise definition is crucial to differentiate genuine cyberterrorism incidents from cybersecurity concerns.

### Cyberterrorism: how it affected the Middle East

Cyberterrorism has recently had a significant negative impact on the Middle East. A variety of cyberattacks have been launched against the area, including assaults on vital infrastructure, the dissemination of propaganda on social media and other online platforms, and the interruption of communication networks. In addition, the region has also been the source of several cyber-attacks, with a few state-sponsored hacking groups operating in the region (Venkatachary, Prasad & Samikannu, 2018). The impact of these attacks has been significant, causing disruption and harm to individuals and societies in the region. The impact of cyber-attacks has been varying depending on a few factors as well the location and infrastructure of countries. However, some countries in the the Middle East region have been the target of many cyber-attacks and have experienced significant disruption as a result. Iran (Rudner, 2013) has been the subject of several cyberattacks, including the Stuxnet worm that disrupted the nation’s nuclear program and the Shamoon malware that erased data from tens of thousands of computers at Saudi Arabia’s Aramco and Qatar’s RasGas. Several cyberattacks have also targeted Saudi Arabia (Elnaim, 2013), notably the Shamoon virus and the “Cutting Sword of Justice” breach, which were directed at the nation’s infrastructure to produce natural gas and oil. Tens of thousands of computers at the Qatari natural gas business RasGas and the Saudi Arabian oil major Aramco had their data destroyed by the Shamoon malware. The attack was believed to have been carried out by Iranian hackers while a group calling itself the “Cutting Sword of Justice” hacked into the computer systems of Saudi Arabian oil and natural gas company Saudi Aramco and released sensitive data online. The group claimed to be protesting Saudi Arabia’s foreign policy and human rights record.

The United Arab Emirates has also been the target of several cyber-attacks, including the “Hack the UAE” campaign (Shires, 2020), which targeted government websites, and the “Sea Turtle” campaign, which targeted several organizations in the country. In 2017, it was discovered that a group of hackers known as “Sea Turtle” had been conducting cyber espionage campaigns against several organizations in the UAE and other countries in the Middle East. The group was believed to be state sponsored (Neagu & Savu, 2019). In 2017, the UAE was targeted by the advanced persistent threat group belonging to DarkHotel, which was believed to be operating out of North Korea. The group targeted a few organizations in the UAE with malware (Alrawi et al., 2021). On 29 December 2022, a hacker downloaded private code repositories using limited swiped employee tokens, however neither Slack’s main codebase nor any client data were contained in the repositories. Slack is one of the most popular workplace communication applications as a result. The hack may have been carried out by an external threat actor, as the owning authority said there was no impact on its code or service as they immediately invalidated those stolen tokens and that the unauthorized access did not stem from a weakness intrinsic to the business. On December 4th, 2023, a data gathering sale including more than 200 million Twitter profiles began. A 59 GB RAR bundle containing the stolen material was made public. The scrapers utilizing earlier data collections were able to compromise the vulnerable API. Microsoft Azure services were susceptible to server-side request forgery (SSRF) attacks on January 17, 2023, due to four vulnerabilities. Azure Functions, Azure Machine Learning, and Azure Digital Twins were among the services offered. If these SSRF flaws had gone unpatched, they might have had a big effect on Microsoft Azure Services. These vulnerabilities were mitigated because of Microsoft’s quick response, which was done before they could do any significant harm (Onat et al., 2022).

### cyberterrorism: how it affected the East Asia

Eastern Asia is one of those regions that have been heavily affected by Cyberterrorism. As a result of inter-regional and intra-regional competition, the prominent countries of East Asia have been indulged in a few cyber-attacks. There have been several cyber-attacks on China in recent years. A wave of cyberattacks known as “Titan Rain” in the middle of the 2000s attacked various American government and military institutions (Taddeo, 2017) as well as businesses in a few other nations. The attacks were believed to have been carried out by hackers based in China. In 2009, it was discovered that a group of hackers had compromised the computer systems of a few organizations in China, including government agencies and embassies. The group, known as “GhostNet” was believed to be based in China (Ghose et al., 2019). It was uncovered in 2015 that China has been launching massive denial-of-service assaults against websites in other nations using a device known as the “Great Cannon”. It was thought that the attacks were a retaliation for criticism of China’s human rights record. Overall, these examples show that China has been the target as well as the base for a few cyber-attacks. Japan has also been the target of cyber warfare over the passage of years. Several Japanese websites, including the government website, were subjected to a series of denial-of-service assaults in 2014 by a gang of hackers going by the name of “Lizard Squad” (Lunsford & Boahn, 2015). In 2015, a group of hackers launched the “OpJapan” campaign, targeting several Japanese websites and organizations. The organization asserted that they were in opposition to Japan’s participation in the Trans-Pacific Partnership trade deal. In 2017, the “WannaCry” ransomware attack affected businesses and government agencies in more than 150 countries, including Japan. The attack was estimated to have cost businesses billions of dollars (Chow, Yau & Li, 2015).

Korea has had some instances of its own when it comes to falling under the radar of cyberterrorism. In 2013, a series of cyber-attacks known as “Dark Seoul” targeted the computer systems of banks and media organizations in South Korea (Marpaung & Lee, 2013). The attacks were believed to be the work of North Korean hackers. The Sony Pictures Entertainment computer systems were breached in 2014 (Afful-Dadzie et al., 2016), which resulted in the disclosure of confidential information and the postponement of the release of the film “The Interview,” which was critical of North Korean leader Kim Jong-un. The incident was attributed to North Korea by the US authorities. In 2017, the “WannaCry” ransomware attack (Kao & Hsiao, 2018) affected businesses and government agencies in more than 150 countries, including South Korea. The attack was estimated to have cost businesses billions of dollars. Some prominent instances of cyberterrorism in Hong Kong include the “GhostNet” attacks, Operation Aurora, and Tibetan Sun. In 2009, it was discovered that a group of hackers had compromised the computer systems of several organizations in Hong Kong, including government agencies and embassies. The group, known as “GhostNet,” was believed to be based in China. In 2010, a series of cyber-attacks known as “Operation Aurora” (Kim, Kim & Park, 2014) targeted several companies in Hong Kong and other countries, including Google. The attacks were believed to be the work of hackers based in China. In 2018, it was discovered that a group of hackers had compromised the computer systems of the Tibetan government-in-exile and other Tibetan organizations. The group, known as “Tibetan Sun,” was believed to be based in China (McVey, 2015). Therefore, it can be observed that just like the Middle East, East Asia has also been the victim of many cyberterrorisms acts over the years and these threats continue to rise in the future as well.

### Cyberterrorism: a global cyber-massacre

As this study discussed in the previous sections about the role and impact of cyberterrorism on Middle East and East Asia, the rest of the world has also fallen prey to this vicious act many a times that makes cyberterrorism a global massacre. USA, one the strongest economic and defense states fell victim to cyber-invasion on several occasions. In 1998, a hacker named “Lozano” launched a series of cyber-attacks on several US government websites, including the websites of the Department of Defense and the US Air Force. In 1999, a series of cyber-attacks known as “Moonlight Maze” targeted several US government agencies, as well as universities and research institutions. The attacks were believed to be the work of Russian hackers (Dawson, 2015). In 2016, Russian hackers were found to have targeted the campaign of Hillary Clinton and hacked the email accounts of Democratic Party officials, leading to the release of sensitive information through WikiLeaks (Nakashima, 2016). Some experts believe that these attacks may have influenced the outcome of the election. In 2020, it was discovered that a group of hackers had compromised the software of SolarWinds, a major provider of IT management software, to gain access to the networks of SolarWinds’ customers. The attack affected several government agencies in the United States, leading to concerns about the integrity of the US government’s systems and the potential for further damage.

The United Kingdom has had its share of numerous cyber incidents. In 2007, a series of cyber-attacks targeted the computer systems of Estonian government agencies, banks, and media outlets. The attacks were believed to be the work of Russian hackers. In 2012, a group of hackers known as “Darkleech” targeted the websites of a few UK businesses, including the Daily Mail and the BBC. The group was believed to be based in Russia (Radhakrishnan, Menon & Nath, 2019). In 2018, the UK government accused Russia of carrying out a cyber-attack on the country’s foreign office and other government agencies. Russia has been always in the news whenever any incident of cyberterrorism is reported anywhere in the world. The reason for this is the involvement of Russian hackers and terrorists behind some of the major cyber-attacks that have happened around the globe. Russian hackers were thought to have carried both the Moonlight Maze assaults in 1999 and the Estonia cyberattacks in 2007. In 2018, the UK government accused Russia of carrying out a cyber-attack on the country’s foreign office and other government agencies (Lam, 2018). Australia has also been dealing with this act along with other nations. In 2018, the Australian Cyber Security Centre was targeted by a cyber-attack. The attack was believed to be the work of a foreign state-sponsored group. In 2015, a group of hackers launched the “OpAustralia” campaign, targeting several Australian websites and organizations. The group claimed to be protesting the Australian government’s proposed data retention laws (Hardy & Williams, 2014). Some of the vulnerabilities were discovered and patched relatively quickly, while others remained unpatched for a longer period, leaving systems and devices at risk. Zero Day was a Chrome browser zero-day vulnerability. It was found in 2022 and gave hackers the ability to run arbitrary code on a user’s computer by tricking them into visiting a malicious website. Although the flaw was fixed in a subsequent version of Chrome, many users remained at risk until they updated.

**Figure 2:**
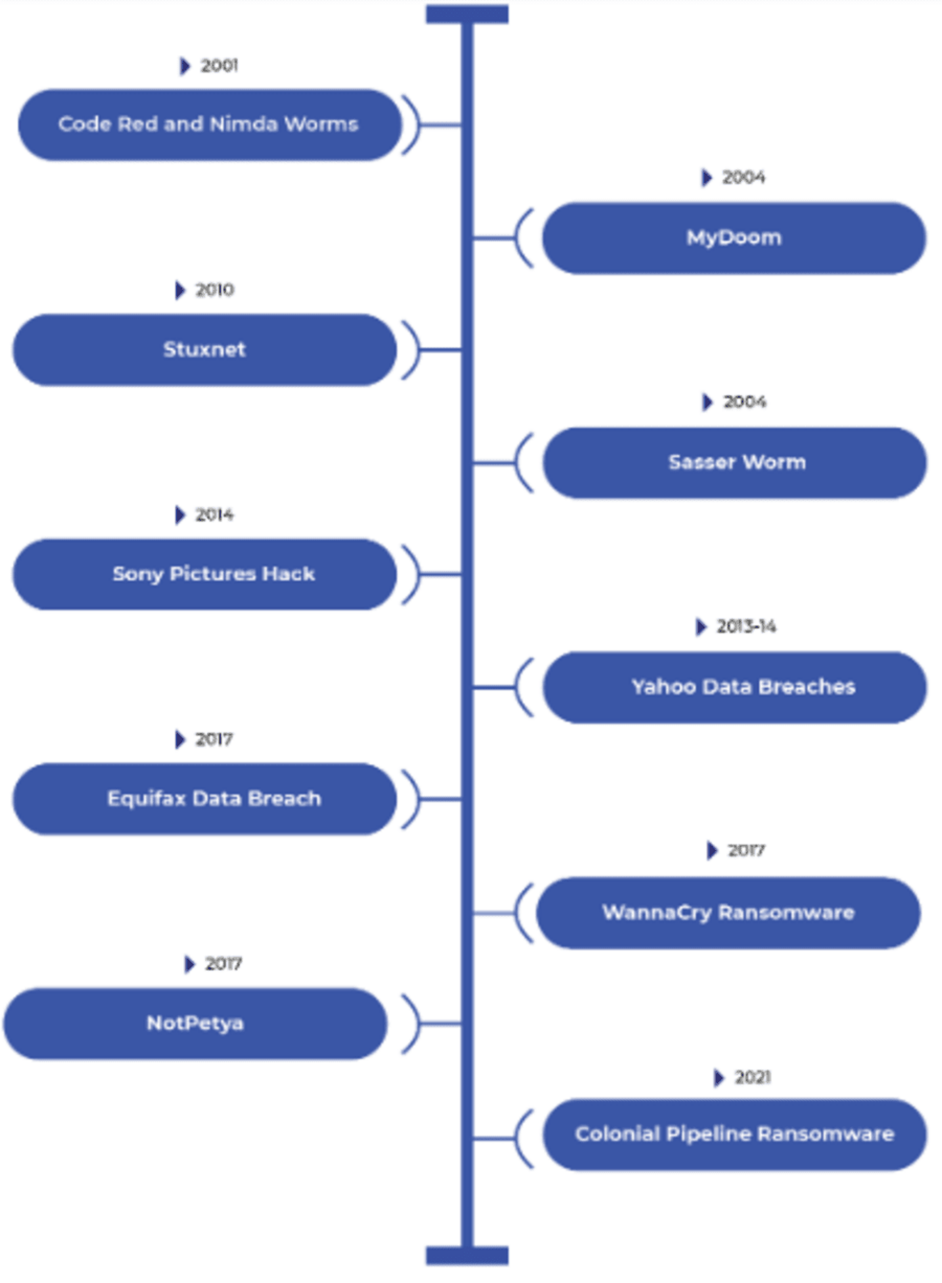
Overview of some of the recent cyberattacks.

Peeking from the conclusions and instances discussed in the previous sections, it can be deduced that Cyberterrorism is a global problem for several reasons. First, the internet is a global network that allows hackers to target individuals and organizations around the world. This means that cyber-attacks can have a global impact, even if they are launched from an individual location. Second, the global nature of the internet makes it difficult to track the origins of cyber-attacks. Hackers can use a variety of techniques to obscure their identity and location, making it difficult to identify the perpetrators of cyber-attacks and hold them accountable. The increasing reliance on the internet and digital technologies has made businesses and governments around the world more vulnerable to cyber-attacks. As increasingly critical systems and infrastructure are connected to the internet, the potential for harm from cyber-attacks increases. Overall, the global nature of the internet and the increasing reliance on digital technologies make cyberterrorism a global problem that requires international cooperation to be addressed. As it intersects with various value systems in various nations, combating cyberterrorism presents a challenging problem. One country’s definition of cyberterrorism may differ from another’s definition of resistance or war. Based on geopolitical factors, historical conflicts, and ideological differences, different contexts are understood differently by cyberterrorism (Tehrani, Manap & Taji, 2013). For instance, in the ongoing conflict in Ukraine, a cyberterrorist aiming for the United States might be viewed as a soldier defending their interests in Russia. It is challenging to develop a consistent, global strategy for addressing cyberterrorism because of the various perspectives and interpretations of this problem. Additionally, there are significant difficulties in combating cyberterrorism related to cross-border enforcement. Because cyberspace has no borders, cyberterrorists can operate from one country while carrying out attacks in another. The speed and anonymity offered by the digital world are difficult for traditional legal systems and jurisdictional boundaries to keep up with. To identify, investigate, and prosecute cyberterrorists, international cooperation becomes crucial. However, ineffective cross-border enforcement efforts are hampered by disparities in legal systems, political unrest, and contrasting priorities. To effectively combat cyberterrorism and hold perpetrators accountable, it is essential to close these gaps and build strong international partnerships. The section below provides an overview of some of the cyberattacks that happened in recent years across the globe. Figure 2 gives a quick look of these cyberattacks.

**Code Red and Nimda Worms (2001):** Code Red and Nimda were two significant worms that targeted Microsoft IIS web servers in 2001. Code Red exploited a vulnerability to deface websites, while Nimda was a multi-vector worm that spread through various means, causing widespread disruptions (Sharma, 2011).

**MyDoom (2004):** MyDoom was a widespread email-based worm that carried a payload designed to launch DDoS attacks against various websites. It was one of the fastest-spreading worms at the time (Chau, 2007).

**Sasser Worm (2004):** Sasser was a computer worm that exploited a vulnerability in Windows operating systems. It caused widespread infections and system instability (Labir, 2004).

**Stuxnet (2010):** Stuxnet was a highly sophisticated worm designed to target industrial control systems, particularly those used in Iran’s nuclear facilities. It was the first known worm specifically developed for cyber-espionage and sabotage. The primary target was Iran’s nuclear program, with a specific focus on centrifuge controls. It caused physical damage to Iran’s nuclear infrastructure and demonstrated the potential for cyberterrorism to have real-world, destructive consequences. It marked a significant shift in the landscape of cyber threats (Collins & McCombie, 2012).

**Yahoo data breaches (2013 and 2014):** Yahoo experienced two massive data breaches that exposed the personal information of billions of users. The breaches were disclosed years later, leading to significant consequences for the company (Whitler & Farris, 2017).

**Sony pictures hack (2014):** Sony Pictures Entertainment was the target of a cyberattack by a group calling themselves the “Guardians of Peace.” The attack resulted in the leakage of sensitive corporate data and unreleased films, causing significant damage to the company. The primary target was Sony Pictures Entertainment. The attack had severe consequences for the company, including financial losses, reputational damage, and legal implications. It also raised concerns about the impact of cyberterrorism on the entertainment industry (Ismail, 2017).

**WannaCry ransomware (2017):** WannaCry was a global ransomware attack that exploited a Windows vulnerability. It infected hundreds of thousands of computers in over 150 countries, encrypting data and demanding a ransom for decryption (Mohurle & Patil, 2017).

**NotPetya (2017):** NotPetya was a destructive ransomware attack that initially masqueraded as a ransomware campaign but had a much broader impact. It exploited a vulnerability in a tax software widely used in Ukraine, spreading through software updates to organizations around the world. It primarily targeted Ukraine, affecting government agencies, financial institutions, and critical infrastructure. However, it quickly spread globally, impacting companies like Maersk, FedEx, and Merck. The attack caused widespread disruption and financial losses, particularly for affected multinational companies. It also raised concerns about the potential for cyberterrorism to cause physical harm, given its impact on critical infrastructure (Fayi, 2018).

**Equifax data breach (2017):** Equifax, one of the major credit reporting agencies in the United States, suffered a massive data breach that exposed the personal information of millions of individuals. It had significant implications for affected individuals’ financial security (Primoff & Kess, 2017).

**SolarWinds supply chain attack (2020):** A sophisticated supply chain attack compromised SolarWinds’ software update mechanism, allowing attackers to distribute malware to thousands of SolarWinds customers. The primary targets were US government agencies, including the Department of Homeland Security and the Pentagon, as well as private sector organizations. The attack exposed sensitive government and corporate data, raising concerns about the potential for cyberterrorism to compromise national security and critical infrastructure. It prompted a significant cybersecurity response and diplomatic efforts (Wolff, Growley & Gruden, 2021).

**Colonial Pipeline ransomware (2021):** Colonial Pipeline, a major US fuel pipeline operator, was targeted by a ransomware attack. DarkSide, a cybercriminal group, was responsible for the attack, which involved encrypting the company’s systems and demanding a ransom for decryption. The attack directly affected Colonial Pipeline, disrupting fuel supplies along the East Coast of the United States. The attack led to fuel shortages, panic buying, and significant economic disruption. Colonial Pipeline paid a substantial ransom to regain control of its systems (Dudley & Golden, 2021; Leu et al., 2023).

These are just a few examples of recent high-profile cyberattacks and data breaches. Cybersecurity threats continue to evolve, and organizations and governments are constantly working to enhance their security measures to defend against these and future attacks. It’s important to stay informed about cybersecurity developments and best practices to protect against such threats.

The attribution and prosecution of cyberterrorism incidents pose several challenges, primarily due to the anonymous and cross-border nature of cyber activities. For example, cyberterrorists often operate under pseudonyms or with a high degree of anonymity, making it challenging to identify the actual individuals or groups responsible for the attacks. Malicious actors can deliberately mislead investigators by attributing their attacks to others, complicating accurate attribution. Cyberterrorism incidents can span multiple countries, which raises jurisdictional issues and complexities in coordinating international investigations and prosecutions. Cyberterrorists may compromise and use the infrastructure of third-party entities, making it difficult to trace the source of the attack back to the actual perpetrators. Advanced Persistent Threats (APTs) groups maintain persistent access to systems and cover their tracks, making detection and attribution more challenging. The use of encryption and anonymization tools can obfuscate communications and hide the origin of attacks. Some cyberterrorism incidents may be linked to nation-states, which can complicate the attribution process due to diplomatic and geopolitical considerations. In some cases, countries may be reluctant to share evidence or collaborate in cyberterrorism investigations, hindering the attribution process. The political implications of attributing cyberterrorism to specific state or non-state actors can affect the willingness to prosecute or take punitive actions. Developing and maintaining strong digital forensics capabilities is essential for attribution, but not all countries or organizations have the required expertise and resources. Balancing the need for effective cybersecurity with privacy and civil liberties concerns can be challenging, especially in cases involving surveillance and data collection. Meeting the legal burden of proof in court, especially when attributing an attack to specific individuals or groups, can be demanding and may require substantial evidence. Despite these challenges, efforts are ongoing to improve attribution and prosecution of cyberterrorism incidents. This includes enhancing international cooperation, sharing threat intelligence, developing more sophisticated forensic techniques, and strengthening cybersecurity laws and regulations. Addressing these challenges is crucial for deterring cyberterrorist activities and holding malicious actors accountable (Tehrani, Manap & Taji, 2013).

### Cyberterrorism: preventions and countermeasures

The consequences of a cyber-attack can be severe, so it is important to take measures to fight cyberterrorism since cyberattacks can have severe consequences. There is a potential of economic loss, damage to a country’s reputation and stability, and even the loss of life due to cyberterrorism. There can also be widespread disruptions and chaos when critical infrastructure, such as power grids and hospitals, are disrupted. The security and reliability of technology can also be undermined by cyberattacks, making people uncertain about the security and reliability of their systems (Leu et al., 2023). Taking preventative measures and mitigating the potential consequences of a cyberattack are two of the most important measures individuals and organizations can take to reduce their risks of being victimized by a cyberattack. Here we will discuss the preventive measures in the form of sections.

### Role of network and system security against cyberterrorism

Security of networks and systems is a critical component of protecting yourself from cyberterrorism. Cyberattacks are often directed at networks and systems to gain access to sensitive information or disrupt operations, which is often the goal of cyberattacks. Organizations can reduce the risk of these types of attacks by implementing effective security measures and reducing the consequences of these attacks by implementing these measures. Security of networks and systems can only be achieved by combining technical controls, such as firewalls and antivirus software, with non-technical controls, such as employee training and incident response plans. Technical controls assist in preventing unauthorized access to networks and systems, whilst non-technical controls serve to guarantee that staff are aware of the importance of cybersecurity and are informed of what to do in the case of an attack (Leu et al., 2023). To ensure that their security measures remain effective against the changing threats landscape, it is imperative that organizations regularly review and update their security measures. The measures taken to address these vulnerabilities may include implementing innovative technology, such as intrusion detection systems and network monitoring tools, and conducting regular security assessments to identify and address potential vulnerabilities.

It is also important for organizations to have a plan in place for responding to a cyberattack, which should include identifying key personnel and establishing clear channels of communication in the event of a cyberattack. There are several network and system security measures (Klein, 2015) that can be adopted to prevent cyberterrorism such as, ensuring the use of strong, unique passwords and enabling two-factor authentication, keeping all the software and security system up to date with latest security patches, using authenticated and strict firewalls, implementing intrusion systems that may alert administration of the potential breach, utilizing a trusted antivirus software, conducting regular security assessments to identify network and system vulnerabilities, implementing access control mechanisms to limit the access of unauthorized users towards sensitive data, having a plan in place in case of a cyber-attack, making sure that employees are educated about cybersecurity best practices, such as not sharing passwords and not clicking on suspicious links etc.

### Cyberterrorism: legal explication and procedures

Global legal systems have been significantly impacted by cyberterrorism. The exploitation of information and communications technologies (ICT) by terrorists, especially the Internet and new technologies that allow for anonymous communication, is a growing worry. An all-encompassing cybersecurity strategy is being developed by the UN Office of Counterterrorism. While some nations have passed national anti-cyberterrorism legislation (*e.g.*, Kenya’s Section 33 of the Computer Misuse and Cybercrimes Act of 2018 and Pakistan’s Section 10 of the Prevention of Electronic Crimes Act of 2016), cyberterrorism is not expressly forbidden by international law. The concept of cyberterrorism has permeated China’s expansive national security agenda, which aims to thoroughly manage, regulate, securitize, and monitor its cyber sovereignty, as demonstrated by Chinese legislation, policies, and judicial practice. This indicates that China has adopted a comprehensive strategy to manage and govern its cyberspace to safeguard its interests in national security (Khater, 2023). Cyberterrorism has a wide range of effects on the US legal system, including those on the criminal justice system, national security, the economy, and civil liberties. The threat that cyberterrorism poses to conventional investigation techniques is one of the most important effects it has on the US legal system. It can be challenging to identify and apprehend those responsible for cyberattacks since they frequently come from people or organizations spread throughout the globe. The investigation and prosecution process are further complicated by the fact that cyberattacks can be launched from any location with an internet connection. As a result, the legal system has had to create fresh approaches to investigating and trying cases of cyberterrorism. To prevent cyberterrorism, the United States has implemented a few legal measures and laws. For instance, the Computer Fraud and Abuse Act (CFAA) of 1986 makes several computer-related offences, like hacking and unauthorized access to computer systems, illegal. Following the 9/11 attacks, Congress passed the Patriot Act, which increased government surveillance capabilities and permitted the gathering of electronic communications data (Wei, 2022).

**Figure 3:**
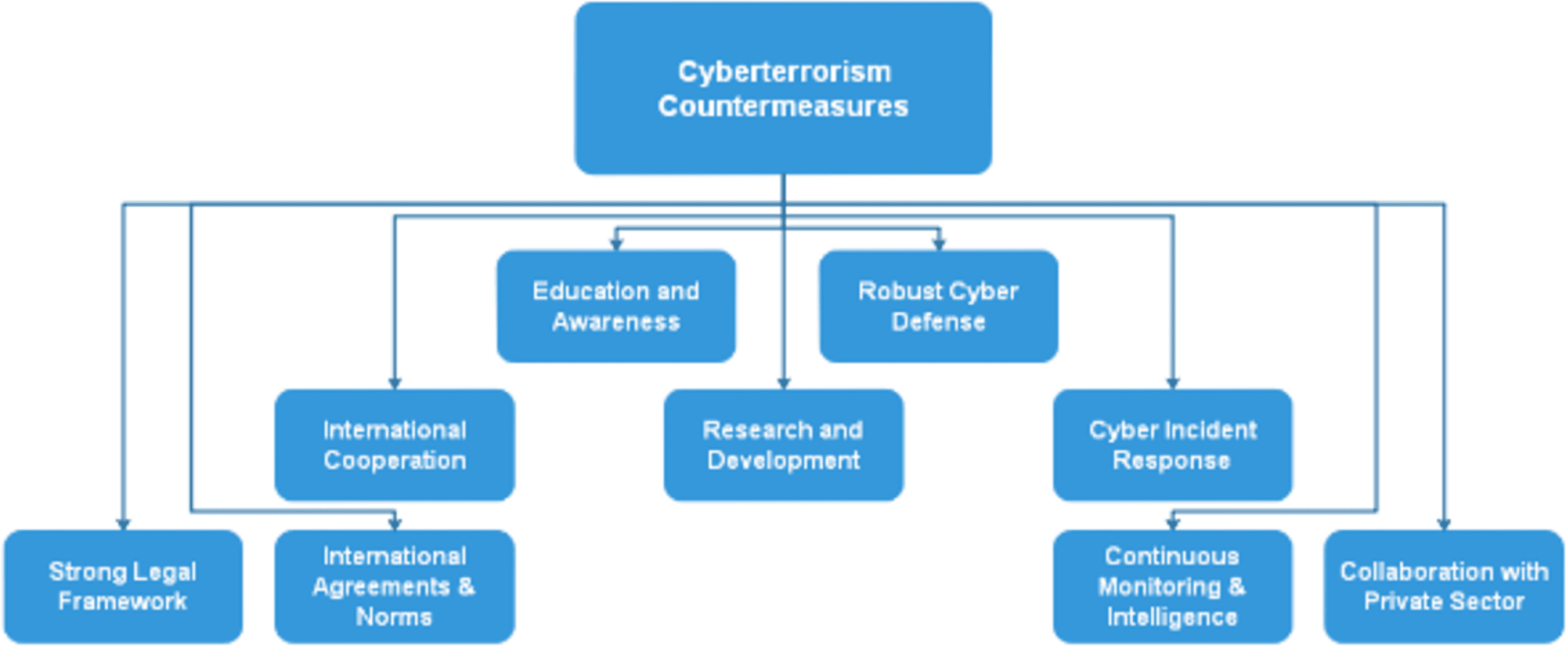
Overview of some of the countermeasures effective against cyberterrorism.

To address cybersecurity and cybercrime, Russia has implemented several laws. The “Yarovaya Law,” which mandates communication carriers to preserve user data for up to three years and give the FSB access to this data, was passed by the State Duma (Russia’s lower house of parliament) in 2016. The law’s opponents claim that it infringes users’ privacy and gives the government excessive authority to track and regulate online activities. Russia has also entered into alliances and agreements of cooperation with other nations. To increase cooperation in avoiding cyberattacks and fostering the development of global norms and regulations in cyberspace, Russia and China issued a joint statement on cooperation in the field of international information security in 2020. Cyberterrorism has been criminalized internationally through a variety of legal means, including legislative, regulatory, and law enforcement activities. The creation of international legal frameworks that offer direction on the prevention and prosecution of cybercrimes has been a significant undertaking. As an illustration, the Council of Europe Convention on Cybercrime, adopted in 2001, offers a framework for harmonizing national legislation on cybercrime and promotes international collaboration in the prevention and prosecution of cybercrime. many nations have created their own laws and rules. The CFAA and the Patriot Act, for instance, give the US government the legal authority to investigate and prosecute cybercrimes, and the NIST has created a framework for enhancing the cybersecurity of critical infrastructure (Baldassarre, 2023). Given that cyberattacks frequently come from outside national borders, international collaboration is also essential in the fight against cyberterrorism. To encourage data exchange and collaborative research, many nations have formed alliances and agreements. For instance, to encourage cooperation on cybersecurity and cybercrime issues, the US and the UK joined the US-UK Cybersecurity Dialogue. In conclusion, a variety of international legal measures have been taken to combat cyberterrorism, including the creation of international legal frameworks, the development of national legislation and regulations, the establishment of specialized units to combat cybercrime, and the encouragement of global cooperation and partnerships. It is expected that nations will continue to develop and adapt legal measures and policies to handle this expanding threat as cyberterrorism develops. Figure 3. shows some of the mainstream countermeasures that a state must adopt to face off the cyberterrorism challenge. These countermeasures can enhance a state’s cybersecurity posture and help protect against cyber threats. Here is a more detailed breakdown of each countermeasure:

**Education and awareness:** Promoting cybersecurity education and awareness among the public, government employees, and critical infrastructure operators is essential to ensure that individuals and organizations are aware of potential threats and how to mitigate them.

**Robust cyber defense:** Developing and maintaining strong cybersecurity measures, including firewalls, intrusion detection systems, and antivirus software, is crucial to prevent and respond to cyberattacks effectively.

**International cooperation:** Collaboration with other countries and international organizations is essential to share threat intelligence, investigate cyber incidents, and develop a coordinated global response to cyberterrorism.

**Research and development:** Investment in cybersecurity research and development is necessary to stay ahead of emerging threats and develop innovative technologies and strategies to protect against cyberterrorism.

**Cyber incident response:** Establishing a well-defined incident response plan helps in effectively managing and mitigating the impact of cyber incidents when they occur.

**Strong legal framework:** Implementing and enforcing cybersecurity laws and regulations provides a legal basis for prosecuting cybercriminals and deterring malicious actors.

**International agreements and norms:** Participating in and promoting international agreements, norms, and treaties related to cyberspace can help establish rules of behavior and cooperation in the digital domain.

**Continuous monitoring and intelligence:** Continuous monitoring of networks and systems, along with intelligence gathering and analysis, allows for early detection of cyber threats and better decision-making in response to those threats.

**Collaboration with the private sector:** Collaboration with private sector organizations, which often own and operate critical infrastructure, is vital. Public–private partnerships can improve information sharing and enhance cybersecurity measures.

By implementing these countermeasures, states can significantly improve their resilience against cyberterrorism and other cyber threats. It is important to adapt and evolve these measures to address the evolving nature of cyber threats and to stay proactive in safeguarding national security and critical infrastructure.

### Analysis of impact—economic, social, and political

The economic, social, and political consequences of cyberattacks, including cyberterrorism, are far-reaching and profound:

### Economic consequences

Cyberattacks resulted in significant financial losses for affected organizations due to theft, fraud, or business disruption with substantial recovery costs, including cybersecurity investments. When customer data is compromised, or services are disrupted, businesses may experience reputational damage that can lead to a loss of customer trust and reduced revenue. Rising cyber insurance premiums and deductibles have burdened businesses, impacting their operational costs. Cyberattacks on critical infrastructure have disrupted supply chains, affecting production, delivery, and the overall economy.

### Social consequences

Data breaches have led to the exposure of sensitive personal information, eroding individuals’ privacy and potentially leading to identity theft or fraud. The fear and anxiety generated by cyberterrorism attacks have a psychological impact on individuals and society, eroding feelings of security and trust. In some cases, particularly when critical services are disrupted, social unrest has occurred as people became frustrated with the inability to access essential services.

### Political consequences

Cyberattacks on government institutions, defense systems, or critical infrastructure posed a significant national security threat, potentially compromising a nation’s ability to defend itself. State-sponsored cyberattacks or cyber espionage escalated international tensions and strained diplomatic relations between countries. High-profile cyberattacks have led to policy changes, new regulations, and increased government involvement in cybersecurity, which has affected businesses and individuals. Failures to protect against cyberattacks eroded public trust in government and its ability to ensure national security and infrastructure resilience.

### Countering cyberterrorism: global policies and procedures

The threat must be countered no matter how critical it is. Therefore, with the ever-growing threat of cyberterrorism, various global policies and countermeasures have been put in place. As part of China’s efforts to counter the threat of cyberterrorism within its borders, there have been several steps taken (Yagya & Ashurova, 2023). As part of the State Council’s cybersecurity plan released last year, the country outlined measures to strengthen the country’s cyber defenses, such as improving the country’s ability to detect and respond to cyber threats as well as enhancing critical infrastructure protection from cyber-attacks. To protect itself against cyberattacks, China has implemented several technical measures in addition to these efforts. To secure networks and systems, firewalls, intrusion detection systems, and other technologies are used. China has also passed a few cybersecurity rules and laws, such as the People’s Republic of China’s Cyber Security Law, which took effect in 2017. This law’s goals are to safeguard the confidential information of the nation’s residents, as well as the key infrastructure of the federal government and the nation’s overall cybersecurity. Additionally, China has established several cybersecurity agencies and organizations for the purpose of coordinating efforts to combat cyber threats. The National Computer Network Emergency Response Technical Team Coordination Center (CNCERT), which coordinates the response to cyber emergencies, and the Cyberspace Administration of China, which oversees managing the nation’s cybersecurity policy, are two of the organizations in charge of doing so.

Several agencies and organizations within the United States have been charged with addressing the threat of cyberterrorism and protecting against cyberattacks to counter it. Among the responsibilities of the Department of Homeland Security (DHS) is the protection of the nation’s critical infrastructure against cyber threats, as well as the coordination of efforts to combat cyberattacks. Information sharing and incident response are handled by the National Cyber Security and Communications Integration Center (NCCIC), a division of the DHS. Investigations and prosecutions of cybercrimes, including cyberterrorism, are the responsibility of the Federal Bureau of Investigation (FBI) (Qi, Shao & Zheng, 2018). To protect military systems and networks from cyberattacks, the Department of Defense (DOD) has implemented a few cybersecurity initiatives. Several laws and regulations have also been implemented by the United States to address cybersecurity. Among them are the Cybersecurity Act of 2015 (Cunningham, 2021; Tran, 2016) and the Cybersecurity and Infrastructure Security Agency Act of 2018. They establish a framework for sharing information and responding to incidents, as well as strengthening critical infrastructure’s cybersecurity. Russia’s Federal Security Service (FSB) is responsible for investigating and prosecuting cybercrimes, as well as protecting against cyber threats. Russia’s Federal Protective Service (FSO) protects the government’s communications and information systems from cyberattacks (Fischer, 2017). The Federal Service for Technical and Export Control (FSTEC), which oversees cybersecurity, regulates Russia’s key infrastructure industries. Russia has enacted a few laws and regulations that address cybersecurity, in addition to the Federal Law on Information, Information Technologies, and the Protection of Information. Also, it describes how government agencies and companies should respond to cyber threats and how personal information should be secured. [Cyber] risks are described in detail.

Saudi Arabia has established National Cyber Security Center (NCSC) responsible for coordinating efforts to protect against cyber threats and promoting cybersecurity in Saudi Arabia (Hindocha, 2020). Under the National Cybersecurity Authority (NCA), cybersecurity measures are implemented in the country and regulations are complied with. Among other things, Saudi Arabia has a cybercrime law that criminalizes cyberterrorism as well. Individuals and organizations involved in cybercrimes can be investigated and prosecuted under this law. The Saudi Arabian National Cybersecurity Regulations and the Saudi Arabian National Cybersecurity Strategy address cybersecurity. The regulations aim to strengthen the cybersecurity of critical infrastructure and establish a framework for sharing information and responding to incidents. Two of the institutions and organizations that have been formed by Saudi Arabia to combat the effects of cybercrime are the Saudi Arabian Monetary Authority (SAMA) and the Saudi Arabian General Investment Authority (SAGIA), respectively (Alshammari & Singh, 2018; Alzubaidi, 2021). UAE’s National Electronic Security Authority (NESA) oversees cybersecurity measures and protects the nation from cyber threats (Al Mazari et al., 2018; Younies & Na, 2020). Several cyber security agencies and organizations have been established to address cyber threats, including the National Crisis and Disaster Management Authority (NCEMA) and National Computer Emergency Response Team (aeCERT) (Grzegorzewski, 2020). In Iran, the National Cyberspace Center (NCC) is responsible for coordinating efforts to protect against cyber threats and promote cybersecurity (Makarova, 2021). Several cybersecurity agencies and organizations have been established in Iran to coordinate efforts to address cyber threats, such as the Center for Strategic Studies on Cyberspace (CSSC) and the National Computer Emergency Response Team (MAHER) (Solgi, Khodaverdi & Poustinchi, 2022).

Similarly, countries like France (Vitel & Bliddal, 2015), Turkey (Atalay & Sanci, 2015), Germany (Lapotnikova, 2019), England (Loveday, 2018), Japan (Christou & Nitta, 2018), Hong Kong (Chang, 2020), Korea (Park, 2021), Belgium (Chang, 2020), Malaysia (Chang, 2020), Indonesia, India (Chang, 2020), Pakistan (Munir & Gondal, 2017), and other countries around the globe have their own dedicated Agencies and institutions that specifically look for any kind of intrusion or invasion from an external source and look to counter it on the spot. Furthermore, all the nations have their own set of rules, mechanisms, laws, and procedures to prevent any kind of inter-regional or intraregional cyberterrorism activity. These preventive measures help in reduction of cyberterrorism up to a massive extent it but, with the passage of time, the technology is getting advanced, and attackers are finding innovative ways of breaching into the security. That is why it is essential to keep monitoring the effects of latest cyber-attacks and proposed countermeasures against them. Table S1 shows an overview of some of the literature works that covered the concept, worldwide causes and countermeasures of cyberterrorism that we have discussed in the previous sections.

### Evolving nature of cyberterrorism—a forward-looking perspective

The evolving nature of cyberterrorism is marked by ever-changing tactics, new attack vectors, and shifting motivations. Current trends, emerging threats, and potential future scenarios highlight the need for continuous vigilance and adaptation in the realm of cyberterrorism. Nation-states are increasingly involved in cyberterrorism, using advanced tools and techniques to further their political and geopolitical goals. These state-sponsored cyberterrorist acts could include attacks on critical infrastructure, espionage, and disruption of services. The convergence of cyber and physical domains opens the door to more destructive cyberterrorist attacks. Future scenarios may involve targeted attacks on Industrial Control Systems (ICS) and Internet of Things (IoT) devices, potentially causing real-world harm and physical destruction. Cyberterrorist groups are adopting Advanced Persistent Threats (APT)-like tactics, techniques, and procedures to maintain long-term access to systems and stealthily conduct espionage or disruptive activities. Cyberterrorism increasingly involves information warfare, disinformation campaigns, and intellectual property theft. Future scenarios may see the use of deepfake technologies to manipulate information and sow confusion. The threat from insiders who have access to critical systems and sensitive data remains a significant concern. Insiders can facilitate cyberterrorism efforts or engage in malicious acts on their own. Ransomware attacks may become more destructive, with cyberterrorist groups employing encryption methods and demanding larger ransoms. Critical infrastructure could be targeted, causing widespread disruption and potential loss of life. As technologies like 5G, quantum computing, and AI continue to advance, cyberterrorists will exploit these developments to launch more sophisticated and hard-to-detect attacks. The globalization of cyberterrorism means that groups can launch attacks from anywhere in the world, making it challenging to attribute and counter their actions. The motives behind cyberterrorism may continue to diversify, including ideological, political, financial, and even environmental factors. Future scenarios may see eco-terrorism involving cyberattacks on energy infrastructure. Cyberterrorism may extend beyond national borders into the realm of international conflict, with attacks serving as a tool in broader geopolitical disputes (Lim, 0000; Sharma, 2023; Brooks, 2023; Kaspersky, 2023).

Addressing these evolving threats requires a forward-looking perspective, focusing on robust cybersecurity measures, information sharing, international cooperation, the development of effective response strategies, and ongoing investment in cybersecurity research and development. Furthermore, public awareness and preparedness are crucial elements in mitigating the evolving nature of cyberterrorism and its potential consequences.

### Policy recommendations

Enhancing cybersecurity and countering cyberterrorism effectively requires a comprehensive approach that involves governments and organizations. Governments should establish a national cybersecurity strategy that outlines clear objectives, roles, and responsibilities. To strengthen their legal frameworks, they should enact and enforce strong cybersecurity laws and regulations to hold malicious actors accountable. Additionally, cyberterrorism-related offenses should be defined and classified. They should implement protective measures for critical infrastructure, such as energy, transportation, and healthcare systems and conduct regular security assessments and audits. Moreover, governments should collaborate with other countries to share threat intelligence, investigate cross-border cybercrime and participate in international agreements and norms to promote responsible behavior in cyberspace. Similarly, they should work closely with the private sector to improve information sharing, develop best practices, and enhance collective defense. Meanwhile, they should invest in cybersecurity education and training programs to address the shortage of skilled cybersecurity professionals and promote cybersecurity awareness and best practices among the public. They should establish national incident response teams, conduct regular drills to prepare for cyber incidents and develop a clear chain of command for incident response. Finally, sharing of advanced threat intelligence among government agencies, private organizations, and international partners should be encouraged.

As far as organizations are concerned, they should educate employees about cybersecurity best practices, such as strong password management, recognizing phishing attempts and implement regular security awareness training programs. Then, they should implement strong access control mechanisms, grant employees the least privilege necessary for their roles, monitor and audit user activities, enable multi-factor authentication (MFA) for all critical systems and applications to enhance authentication security. Additionally, they should maintain up-to-date software, apply security patches promptly to address vulnerabilities, create a patch management process to ensure timely updates and segment networks to limit lateral movement for attackers and contain potential breaches. Moreover, Security Information and Event Management (SIEM) solutions should be developed to monitor and analyze network activity for early threat detection and incident response. Cyber insurance policies should be purchased to mitigate financial risks associated with cyber incidents. Data encryption and backup techniques should be used to encrypt sensitive data at rest and in transit and to regularly back up data and ensure that backup systems are secure and accessible. Finally, organizations should assess and monitor the cybersecurity posture of third-party vendors and suppliers who have access to your systems or data. For that develop and test an incident response plan that includes steps for containment, eradication, and recovery (IETF, 2007; ISO, 2009).

By following these policy recommendations and implementing these practical steps, governments and organizations can significantly enhance their cybersecurity and resilience against cyberterrorism and other cyber threats. A proactive and collaborative approach is crucial in the ever-evolving landscape of cyberspace.

### Legal and ethical aspects

Legal and ethical considerations surrounding cyberterrorism are critical in addressing the complex challenges posed by acts of cyberterrorism. The international legal framework and national laws and regulations play a central role in defining, prosecuting, and preventing cyberterrorism. For example, the United Nations (UN) has been at the forefront of addressing cyberterrorism through its General Assembly resolutions, which encourage member states to cooperate and develop norms for responsible state behavior in cyberspace (Henderson, 2021). The Tallinn Manual, a non-binding document developed by experts, provides interpretations of existing international law applicable to cyberspace, offering guidance on the legal framework surrounding cyber operations (Pipyros et al., 2018). The Budapest Convention on Cybercrime, also known as the Council of Europe Convention on Cybercrime, is a multilateral treaty aimed at harmonizing laws and enhancing international cooperation in combating cybercrime, which includes provisions related to cyberterrorism (Wicki-Birchler, 2020).

Many countries have established specific laws and regulations related to cyberterrorism, outlining offenses, penalties, and prosecutorial authorities. These laws vary from one jurisdiction to another, but common elements include defining cyberterrorism-related offenses, such as hacking, data breaches, or DDoS attacks, and specifying punishments. Laws often address issues related to jurisdiction, attribution, and extradition, as cyberterrorism can involve actors and activities across borders. Determining the intent and attribution in cyberterrorism cases can be challenging, as it requires clear evidence to establish motive and identify the responsible actors. Balancing security and civil liberties, including privacy and freedom of expression, is an ongoing ethical concern. Measures taken to counter cyberterrorism must be proportionate and respectful of individual rights. Prosecution of cyberterrorists involves legal processes that need to account for digital evidence, chain of custody, and international cooperation. Ensuring accountability for state-sponsored cyberterrorism can be complex, as it may involve diplomatic negotiations, international law, and political considerations. Preventing cyberterrorism involves addressing root causes and vulnerabilities, such as improving cybersecurity, countering radicalization, and promoting international cooperation to deter malicious actors. Upholding human rights is essential in the context of cybersecurity and countering cyberterrorism, as overly broad or invasive measures can infringe on individual freedoms. In summary, the legal and ethical considerations surrounding cyberterrorism involve a complex interplay of international legal frameworks, national laws, and ethical dilemmas. The pursuit of a balance between security and individual rights, as well as the development of effective legal mechanisms for prosecution and prevention, remains a challenge in addressing the evolving nature of cyberterrorism (Tehrani, 2017).

### Future work

The future research on cyberterrorism should encompass various critical areas to provide a comprehensive understanding of this evolving threat landscape. Firstly, investigating perceptions and awareness among diverse stakeholders, such as individuals, organizations, and government agencies, will shed light on the level of preparedness and recognition of cyberterrorism risks. Secondly, analyzing the impact of cyberattacks on different sectors, including finance, healthcare, and critical infrastructure, will offer valuable insights into the potential consequences and vulnerabilities that need to be addressed. Additionally, evaluating the effectiveness of existing countermeasures and strategies, along with identifying emerging technological trends, can help inform policymakers and security experts on the best practices to mitigate cyber threats effectively. Another crucial aspect of survey-based research is the examination of international cooperation and collaboration in combating cyberterrorism. As cyberattacks transcend national borders, understanding the extent of information sharing, joint efforts, and international treaties can highlight areas where global cooperation can be strengthened. Furthermore, investigating the legal and policy frameworks of various countries in response to cyberterrorism is essential to identify gaps and inconsistencies, enabling the formulation of harmonized and effective cyber laws. Such research can also delve into public perception and media influence, as understanding how the public perceives cyberterrorism can impact response strategies and public policies.

Moreover, exploring the psychological impact of cyberterrorism on society is critical to understanding the fear and anxiety generated by such threats. Survey-based research can help assess the emotional and psychological responses of individuals and communities, informing strategies to alleviate distress and enhance resilience. Lastly, an essential aspect of this research involves anticipating future threats and trends in cyberterrorism. By analyzing historical patterns and emerging technologies, researchers can contribute to proactive cybersecurity measures and policy development. In conclusion, survey-based research on cyberterrorism should address these multifaceted areas to provide a comprehensive and valuable contribution to the field of cybersecurity and global efforts to combat this complex and rapidly evolving threat.

## Conclusions

Cyberterrorism involves the exploitation of computer and internet-based technologies to commit acts of violence. A variety of methods can be used, including hacking into computer systems to steal sensitive information, spreading malware to disrupt operations, and inciting violence or sowing discord on social media. Financial losses, life losses, reputational damage, and loss of stability can all be the results of cyberterrorism. Global efforts are being made to increase cybersecurity and strengthen resilience of critical systems against these attacks, which is a growing concern for governments and businesses. As a part of these efforts, non-technical and technical measures, such as staff training and incident response plans, are being introduced. Examples of technological measures include firewalls and antivirus software. However, despite these efforts, cyberterrorism has continued to be a significant threat. This is due to the ever-changing nature of the internet, as well as the ever-increasing reliance on technology across all facets of society because of the ever-increasing use of technology. Both factors contribute to the continued existence of cyberterrorism. To protect themselves from this threat, individuals and organizations must remain vigilant and update their security measures regularly to protect against it. Our survey analyzing types of cyberattacks, sectors targeted, repercussions, and countermeasures reveals a diverse landscape of threats, with phishing, malware, and DDoS attacks being common, impacting sectors such as finance, healthcare, and government most severely. Our findings also highlight the economic and reputational damage incurred by organizations and governments, as well as the need for robust cybersecurity strategies. However, our limitations include challenges in obtaining comprehensive data due to underreporting, the dynamic nature of cyber threats that evolve rapidly, and potential selection bias based on the sources surveyed. Additionally, measuring the effectiveness of countermeasures remains a complex endeavor, and their adaptation is hindered by resource constraints and evolving attacker tactics. Since cyberterrorism is an ever-evolving issue, future research could focus on finding the impact of certain policies developed by certain countries over cyberterrorism.

## Supplemental Information

10.7717/peerj-cs.1772/supp-1 Supplemental Information 1 Overview of some of the literature works that covered the concept, worldwide causes and countermeasures of Cyberterrorism

## Additional Information and Declarations

### Data Availability

The following information was supplied regarding data availability: This is a literature review.
