# Supplementary material for: Cyberterrorism as a global threat: a review on repercussions and countermeasures
Source: PeerJ Comput Sci. 2024 Jan 15;10:e1772. doi: 10.7717/peerj-cs.1772 (PMC10803091; doi:10.7717/peerj-cs.1772)
Supplement: Supplemental Information 1 [file peerj-cs-10-1772-s001.pdf]

**Table 1: Overview of some of the literature works that covered the concept, worldwide causes and countermeasures of Cyberterrorism**

| Ref. | Year | Source | Region | Aim |
|---|---|---|---|---|
| [20] | 2018 | Journal of Cyber Security Technology, Taylor & Francis Online | India | Impact of cyberterrorism on India's energy sector |
| [21] | 2013 | International Journal of Intelligence and Counter-Intelligence, Taylor & Francis Online | Canadian Security Intelligence Service (CSIS), Canada | Impact of Cyberterrorism on National security and infrastructure of Canada |
| [22] | 2013 | Information and Knowledge Management, IISTE | Saudi Arabia | Cyber-crimes spread through the increased use of internet in all real-world aspects in Saudi Arabia |
| [23] | 2020 | Texas National Security Review | USA | Political scandals, leaks and operations between USA and its allies in Persian Gulf |
| [24] | 2019 | Knowledge Horizons. Economics, EconPapers | Romania, Southeastern Europe | Impact of cyberterrorism on shipping, transportation, production lines and critical infrastructure of East Asia & North Africa |
| [25] | 2021 | Proceedings of the 30th USENIX Security Symposium | Georgia, USA | Analyzing impact of Cyberterrorism worldwide and forecasting malware capabilities from memory images |
| [26] | 2022 | Behavioral Sciences of Terrorism and Political Aggression | Scranton, Pennsylvania | To identify determinants of fear in cyberterrorism compared to traditional terrorism and terrorist attacks using the 2018 American Fears Survey data |
| [27] | 2017 | Minds and Machines, Springer | Oxford Internet Institute, University of Oxford, Oxford, UK | Covering the strategies and normalizations approved by G7 countries against Cyber crimes |
| [28] | 2019 | 2019 Spring Simulation Conference, IEEE Xplore | Tucson, Arizona, USA | Multimodal graph analysis of cyber-attacks |
| [29] | 2015 | Grey Literature, ICTN Term Paper | USA | Analyzing the attacking strategy of Lizard attacks on Sony PlayStation and Microsoft X-Box |
| [30] | 2015 | International Conference on Critical Infrastructure, Springer | Hong Kong, China | Impact of cyberterrorism on political events happening inside Hong Kong and China |
| [31] | 2013 | Indonesian Stud. Assoc. Korea | Korea | Detailed analysis of Dark Seoul cyber-attack over banks and broadcasting services in South Korea |

| | | | | |
|---|---|---|---|---|
| [32] | 2016 | ICCWS 2016 11th International Conference on Cyber Warfare and Security Boston Massachusetts USA | Massachusetts USA | Detailed study on parameters and reasons for 2014 Sony pictures hack |
| [33] | 2018 | 2018 20th International Conference on Advanced Communication Technology (ICACT), IEEE | New Taipei City, Taiwan | Detailed study on parameters and reasons for 2017 WannCry ransomware attack that targeted Microsoft windows running devices |
| [34] | 2014 | Mobile, Ubiquitous, and Intelligent Computing, Springer | Korea | Worldwide analysis of cyber-attacks and security measures |
| [35] | 2015 | Grey Literature, ProQuest | Utica, New York, USA | Analyzing reasons, instances and evolution of worldwide cyber-attacks and their countermeasures |
| [36] | 2015 | Grey Literature, Google Books | St. Louis, USA | Study of emerging dangers and defenses against them in the realm of cybercrime and cyberterrorism |
| [37] | 2016 | The Washington Post | USA | Deep look inside Russian hackers Cyberterrorism attempt on the Democratic party and information theft regarding Trump |
| [38] | 2019 | TENCON 2019 - 2019 IEEE Region 10 Conference, IEEE | Calicut, India | Detailed survey on zero-day malware attacks and methods developed for their detection |
| [39] | 2018 | Grey Literature, HeinOnline | USA | Analyzing the aspects responsible for Russian hacker’s attack on 2016 US Presidential elections |
| [40] | 2014 | Cyberterrorism, Springer | Sydney, NSW, Australia | Analyzing the concern, impact, definitions, and countermeasures for Cyberterrorism on Commonwealth nations |
| [41] | 2013 | Computer Law & Security Review, Elsevier | Malaysia | The study of Cyberterrorism as a global challenger and debating the need of a global response against this act of multi-jurisdictional crime |
| [51] | 2017 | International Journal of Science and Research (IJSR) | Malaysia | The detailed study of cyberterrorism, its strategies, stages and how it puts global infrastructure at risk |

| | | | | |
|---|---|---|---|---|
| [52] | 2015 | Journal of Strategic Security, JSTOR | Florida, USA | The in-depth analysis of cyberterrorism, including its tactics, phases, and how it endangers the world's infrastructure |
| [53] | 2023 | : International Journal of Sociotechnology and Knowledge Development (IJSKD) | Saudi Arabia | To review and analyze the legal framework for safeguarding cyberspace from terrorist acts using various approaches. It suggest the necessity of international efforts and the establishment of a legal treaty to effectively combat cyberterrorism, protect global security and stability. |
| [54] | 2022 | Computer Law & Security Review, Elsevier | United Kingdom | Critical analysis of China's legal remedies to cyberterrorism |
| [55] | 2023 | MDPI | Firenze, Italy | The research explores the use of cyberspace by religious fundamentalist groups for terrorism-related activities and emphasizes the need for a comprehensive European legal response and global collaboration to address the growing security challenges posed by cyberspace. |
| [56] | 2021 | Proceedings of Topical Issues in International Political Geography | Russia | The research explores the interaction of Russia and China within the SCO, focusing on their roles, projects, and Sino-Russian relations in the organization. |
| [57] | 2018 | Computer Law & Security Review, Elsevier | China | Understanding the Cybersecurity law formulated by China, its effectiveness, reasons that led to its formulation and drawbacks |
| [58] | 2021 | Grey Literature, ProQuest | Minnesota, USA | Understanding deterrent and reaction enhancements for a large-scale cyberattack |
| [59] | 2017 | Defense Technical Information Center | Library of Congress, Washington DC | Survey of Cyberterrorism as a global challenge, its effects, remedies and countermeasures |
| [60] | 2020 | Third Way, JSTOR | USA | A peek at the latest policies, law enforcement strategies, implementations and budget cuts adopted by USA since 2020 against Cyberterrorism |
| [61] | 2016 | Grey Literature, HeinOnline | USA | Detailed analysis of Cybersecurity Act, 2015 |

| | | | | |
|---|---|---|---|---|
| [62] | 2020 | Journal of Crime and Justice, Taylor & Francis | Georgia, USA | Covering previously proposed methodologies in cybercrime research and debating on new direction being followed since 2019 |
| [63] | 2021 | Ural Symposium on Biomedical Engineering, Radioelectronics and Information Technology (USBEREIT), IEEE | Yekaterinburg, Russia | The article examines computer attack (CA) implementation from the intruder's perspective, identifying stages like goal achievement, theoretical and practical training, and the factors influencing the choice of CA method. It also outlines the conditions for terminating a CA by the intruder or the organization. |
| [64] | 2021 | Heliyon, Elsevier | Saudi Arabia | Covering the impact of Cyberterrorism on Saudi Arabia and investigating the awareness level with the passage of time among the residents |
| [65] | 2018 | Archives of Business Research, Academia | Saudi Arabia | This research examines Saudi Arabia's semi-preparedness to defend against cyber-crimes and suggests improvements, including strengthening the anti-cybercrimes law and promoting the cyber-security industry. |
| [66] | 2018 | Cyber Security and Threats: Concepts, Methodologies, Tools, and Applications | Saudi Arabia | This paper identifies common features in cyber terrorism, proposes effective mitigation strategies, and develops taxonomies for definition, targets, and risk factors. |
| [67] | 2020 | Journal of Financial Crime, Emerald Insight | UAE | Covering the impact of Cyberterrorism on UAE, the impact and effectiveness of anti-cyberterrorism laws on citizens and businesses in UAE |
| [68] | 2022 | International Journal of Political Science (IJPS) | Iran | Analyzing the long-lasting effects of Cyberterrorism on Iran and how this act still poses a threat for the foreseeable future |
| [69] | 2015 | Information & Security: An International Journal | France | This article provides an overview of French cybersecurity and defense policies, highlighting efforts to catch up with strategic partners through conceptual reformulation, organizational changes, and increased resources. It focuses on the role of the National Information Systems Security Agency (ANSSI) and the ministries of defense and interior in addressing cybersecurity threats. |

| | | | | |
|---|---|---|---|---|
| [70] | 2015 | Information & Security: An International Journal | Turkiye | The article delves into the emergence of cyberspace, cyber threats, Turkey's counter-cyberterrorism measures, and the need for international cooperation to strengthen cybersecurity. |
| [71] | 2019 | Journal of Governance and Politics | Russia | The impact of Cyberterrorism on Germany and Russia, their roles behind it globally and the measures they adopted to fight it off |
